# Predictive factors of toxicity of TPF induction chemotherapy for locally advanced head and neck cancers

**DOI:** 10.1186/s12885-021-08128-5

**Published:** 2021-04-07

**Authors:** Maureen Bernadach, Michel Lapeyre, Anne-Françoise Dillies, Jessica Miroir, Melanie Casile, Juliette Moreau, Ioana Molnar, Angeline Ginzac, Nathalie Pham-Dang, Nicolas Saroul, Xavier Durando, Julian Biau

**Affiliations:** 1 Medical Oncology Department, Jean PERRIN Center, 63011 Clermont-Ferrand, France; 2 Clinical Research Division, Delegation for Clinical Research and Innovation, Jean PERRIN Center, 63011 Clermont-Ferrand, France; 3 Clinical Investigation Center, UMR501, 63011 Clermont-Ferrand, France; 4 Radiotherapy department, Centre de Lutte Contre le Cancer Jean PERRIN, 58 Rue Montalembert, 63011 Clermont-Ferrand, France; 5 Clermont Auvergne University, INSERM, U1240, Molecular Imaging and Theranostic Strategies, Jean PERRIN Center, 63011 Clermont-Ferrand, France; 6 Department of Maxillofacial and Plastic Surgery, Estaing University Hospital Center Clermont-Ferrand, 63000 Clermont-Ferrand, France; 7 Department of Otorhinolaryngology - Head and Neck Surgery, Gabriel Montpied University Hospital Center, 63000 Clermont-Ferrand, France

**Keywords:** Head and neck cancer, Induction chemotherapy, TPF, Toxicity, Nutritional status, Hepatic dysfunction

## Abstract

**Background:**

The rate of toxic deaths related to induction chemotherapy in the treatment of locally advanced head and neck cancers is unacceptable and calls into question this therapeutic strategy, which is however highly effective in terms of rate and speed of response. The purpose of the study was to investigate predictive factors of toxicity of induction chemotherapy with docetaxel, cisplatin, and 5-fluorouracil (TPF) in locally advanced head and neck cancers (LAHNC).

**Methods:**

Between June 2009 and December 2017, 113 patients treated consecutively with TPF were included retrospectively. Patients were receiving induction chemotherapy for either an inoperable cancer or laryngeal preservation. For inoperable cancer, induction chemotherapy was proposed to patients presenting either a large tumor with strong symptoms (dyspnea, dysphagia, pain) or a tumor with rapid progression. Risk factors were chosen among the initial patient and tumour characteristics and chemotherapy modalities.

**Results:**

Eighty-nine patients (79%) were male; the median age was 58 years [32–71]. Sixty-nine (61%) patients were treated for inoperable cancer and 44 (39%) for laryngeal preservation. 45% had stage IVa cancer, 28% stage III and 25% stage IVb. Sixty percent of patients had a partial response after TPF, 22% had a complete response, 12% were stable, 5% were progressing, and 1% had a discordant response. Thirty-four patients (30%) received enteral feeding during induction chemotherapy with TPF. The possibility of oral feeding without a tube was predictive of a better response (*p* = 0.003). Seven (6%) patients died during TPF. There was an increased risk of death with preexisting liver dysfunction (liver dysmorphia on imaging or decrease prothrombin rate) (*p* = 0.032). There was an increased risk of grade ≥ 3 infection if an enteral feeding occurred during the period of induction chemotherapy (p = 0.03).

**Conclusions:**

TPF induction chemotherapy had an 82% objective response rate with 6% toxic deaths. Nutritional status and the presence of hepatic dysfunction are significant risk factors to be taken into account in therapeutic decisions.

**Supplementary Information:**

The online version contains supplementary material available at 10.1186/s12885-021-08128-5.

## Background

Sixty percent of head and neck squamous cell carcinomas (HNSCC) are diagnosed at locally advanced stages [1]. Until the early 1990s, local treatments (surgery and/or radiotherapy [RT]) were the key components of treatment for locally advanced head and neck cancers (LAHNC) [2, 3] with a high rates of relapse and morbidity [4, 5]. To improve cure rates and functional outcomes, chemotherapy has been integrated into various strategies (concurrent radiochemotherapy, induction chemotherapy and a combination of both) [6–13]. These strategies have been applied in patients with non-operable cancers [6–11, 14] and in patients with resectable disease who are candidates for organ preservation [9, 12, 13, 15–17].

Induction chemotherapy combining cisplatin-5-fluorouracil (PF) followed by RT was initially approved for organ preservation in locally advanced, operable laryngeal squamous cell carcinomas (SCC) requiring total laryngectomy [17, 18]. In 2006, the GORTEC 2000–01 [19] trial demonstrated the superiority of the docetaxel-cisplatin-5-fluorouracil (TPF) combination to the PF induction regimen in the management of these laryngeal SCC eligible for an organ preservation strategy. For inoperable LAHNSCC, a large meta-analysis [11] compared TPF to PF induction, also showing TPF’s superiority. Thus, TPF induction chemotherapy, already used in laryngeal preservation strategies, has been extended to all inoperable LAHNSCC with large tumour (T3 - T4) or lymph node extension (N2c - N3) with high risk of micro-metastases, without any formal proof of its superiority or non-inferiority compared to the standard treatment of concomitant radiochemotherapy.

The exact place of TPF induction chemotherapy for LAHNSCC over standard concomitant radiochemotherapy [10] has been questioned by several studies [20–25]. The National Comprehensive Cancer Network (NCCN) guidelines include induction chemotherapy with TPF followed by RT alone or by radiochemotherapy [26] as a validated treatment option. Indeed, TPF induction chemotherapy continues to be used due to a high response rate and an unequalled rapidity of response, often allowing a rapid regression of severe symptomatology (dyspnea, dysphagia, pain…).

However, TPF induction chemotherapy introduces a high risk of severe toxicities. These toxicities, in patients with many co-morbidities, can be fatal, with death rates ranging from 0 to 6.7% [8, 20–23, 27]. In addition, 20–30% of patients starting induction chemotherapy do not receive all of the radiotherapy +/− concomitant chemotherapy [8, 28, 29]. Thus, this treatment strategy may show better outcomes in better-selected patients with a lower risk of toxicity and better chances of receiving the full course of treatment [21–23, 25, 30, 31]. For now, there are no consensus criteria for selecting these patients. The main aim of this study was to identify predictive factors of toxicity of docetaxel, cisplatin, and 5-fluorouracil (TPF) in locally advanced head and neck cancers (LAHNC), in order to better select patients likely to benefit from this treatment.

## Methods

### Inclusion population and study enpoints

Between June 2009 and December 2017, 113 patients treated consecutively in our institution with TPF induction chemotherapy were analyzed retrospectively. Patients were receiving induction chemotherapy for either an inoperable cancer or laryngeal preservation. For inoperable cancer, induction chemotherapy was proposed to patients presenting either a large tumor with strong symptoms (dyspnea, dysphagia, pain) or a tumor with rapid progression.

According the French legislation, the database has been declared to the French National Commission on Informatics and Liberty. All patients have been informed about the research by a non-opposition letter. They were free to oppose to the used of their personal data for this study. Study ethics approval was obtained on 18 November 2020 (CECIC Rhône-Alpes-Auvergne, Grenoble, IRB 5921).

The primary endpoint of this study was to identify predictive factor of grade ≥ 3 toxicities of TPF induction chemotherapy. Secondary endpoints included the search of predictive factors of treatment-related deaths, predictive factors of overall response rates and overall survival.

### TPF induction chemotherapy

The induction chemotherapy regimen combined docetaxel 75 mg/m^2^ on D1, cisplatin 75 mg/m^2^ on D1 or cisplatin 20 mg/m^2^ from D1 to D4, and 5-fluorouracil 750 mg/m^2^ as a continuous infusion from D1 to D5 every 21 days. Intravenous hydration of 2 to 3 L was administered depending on whether patients were receiving single-dose or fractionated cisplatin. The antiemetic protocol combined aprepitant, corticosteroids, ondansetron and anti-D2. All patients received systematic GCSF as primary prophylaxis in accordance with international recommendations [32]. Premedication with methylprednisolone 50 mg twice daily was given the day before, the day of and the day after docetaxel in order to prevent hypersensitivity reactions, cutaneous adverse reactions and retention syndromes. Systematic dihydropyrimidine dehydrogenase (DPD) deficiency screening was not realized in the study period. According to local recommendations, systematic ciprofloxacine as primary prophylaxis was not realized.

### Treatment following TPF induction chemotherapy

Radiotherapy delivered curative doses on the initial tumor/tumor bed of 60 and 66 Gy for operated patients and 70 Gy for non-operated patients, in fractions of 2 Gy to 2.12 Gy through conformal technique or volumetric modulated arc therapy (VMAT). RT was administered alone or concurrently with chemotherapy, which consisted of carboplatin AUC 1.5/week for 7 weeks or cisplatin 40 mg/m^2^/week for 7 weeks or cisplatin 100 mg/m^2^ W1, W4, W7 or cetuximab 400 mg/m^2^ at D-8 and 250 mg/m^2^/week for 7 weeks.

### Patient follow-up

Acute hematologic and non-hematologic adverse events occurring during induction chemotherapy or radiotherapy were evaluated according to the Common Terminology Criteria for Adverse Events (CTCAE) version 4.0.

Responses obtained after TPF and two to three months after the end of radiation therapy were also reported. Tumour response was evaluated clinically by nasofibroscopy and/or CT imaging according to the Response Evaluation Criteria in Solid Tumours (RECIST) 1.1. Patients’ responses included complete and partial response, and treatment failure included patients with progression or no response (stable disease) in order to increase the power of the analyses.

### Statistical analysis

Statistical analyses were performed using R software (v. 3.5.1, R-Project, GNU GPL [33]). The normality of the continuous variables was tested using a Shapiro-Wilk test. For the statistical hypothesis testing, the α risk was fixed to 5%. Initial patient and tumour characteristics and chemotherapy modalities were analyzed in search of predictive factors of toxicity, death, progression-free survival (PFS) and overall survival (OS). Risk factors of toxicity studied were age, gender, alcohol consumption, presence of hepatic dysmorphia, prothrombin level, diabetes, high blood pressure, history of ischemic heart disease, chronic obstructive bronchitis, obliterative arterial disease of the lower limbs, performance status, weight loss before and during induction chemotherapy and receiving enteral feeding during induction chemotherapy indication for laryngeal preservation or inoperable disease and stage of disease.

The methods used were Fisher’s exact test (for the relationship between pairs of categorical variables), the Wilcoxon-Mann-Whitney test (in case of a continuous and a categorical variable), and a Cox model (for time-to-event data). Statistical significance in the Cox model was assessed using the Wald statistic. Multivariate Cox models were fit using a variable selection method based on a LASSO-type penalized regression using all factors with a *p*-value < 0.1 in the univariate analysis.

Survival was defined as the time between diagnosis and death, regardless of cause, and living patients had their conditions at the date of the last follow-up statistically censored. Event-free survival was calculated as the time from the date of diagnosis to the date of progression (local, regional, or distant) or death, with patients’ conditions at the last follow-up being censored if no event had occured. Kaplan-Meier estimates were used for survival rate evaluation, and comparison between survival curves was based on the logrank test.

## Results

### Characteristics of the population

Sixty-nine (61%) had inoperable LAHNC and 44 (39%) were treated in a laryngeal preservation strategy. The patient and tumor characteristics are summarized in Table 1 and Table 2. The median age was 58 years [32–71]. 96% of patients had a World Health Organization (WHO) performance status of 0 or 1. 70% of patients had a history of alcohol and tobacco intoxication. The most frequent tumor sites were the larynx (27%). 92% of cancers were SCC, 7% undifferentiated carcinoma and 1% adenocarcinoma. All tumors were locally advanced with 45% of them classified as stage IVa, 28% stage III and 25% stage IVb. In inoperable tumors, there were one stage II (1%), 9 stage III (13%), 33 stage IVa (48%) and 26 stage IVb (38%). In laryngeal preservation, there were one stage II (2%), 23 stage III (52%), 18 stage IVa (41%) and 2 stage IVb (5%).

**Table 1:** Characteristics of 113 patients treated with TPF^a^ induction chemotherapy for locally advanced head and neck cancer

**Male sex – no. (%)**	89 (79)
**Average age [min-max] (years)**	58 [32–71]
**WHO Performance Index – no. (%)**	
0	56 (50)
1	52 (46)
2	5 (4)
**Alcohol and tobacco use – no. (%)**	
Alcohol and tobacco use	79 (70)
Tobacco	19 (17)
Alcohol	4 (3)
No alcohol and tobacco use	11 (10)
**Comorbidities – no. (%)**	
Hepatic dysfunction	6 (5)
Hepatic dysmorphia	10 (13)
Diabetes	9 (8)
High blood pressure	17 (15)
Ischemic heart disease	2 (2)
Obliterative arteriopathy of the lower limbs	5 (4)
Obstructive pulmonary disease	8 (7)
Stroke	2 (2)
**Weight loss – median [min-max] (Kg)**	−4,1[−28–7,81]
**Serum Albumin – median [IC-95%] (g/l)**	39,6 [37,6-39,8]
**Oral feeding possible at diagnosis – no. (%)**	
Yes	88 (78)
No	25 (22)
**Enteral nutrition during TPF – no. (%)**	
Yes	34 (30)
No	79 (70)

^a^*TPF* docetaxel, cisplatin, 5-fluorouracil

**Table 2:** Tumor characteristics of 113 patients treated with TPF^a^ induction chemotherapy for locally advanced head and neck cancer

**Squamous cell carcinoma – no. (%)**	102 (90)
**Primary disease site – no. (%)**	
Oral cavity	7 (6)
Oropharynx	26 (23)
Nasosinus	11 (10)
Nasopharynx	10 (9)
Hypopharynx	27 (24)
Larynx	31 (27)
Adenopathy without primitif	1 (1)
**Laryngeal preservation – no. (%)**	44 (39)
**Stage – no. (%)**	
II	2 (2)
III	32 (28)
IVA	51 (45)
IVB	28 (25)
**Tumor size – no. (%)**	
T1	3 (3)
T2	15 (13)
T3	52 (46)
T4	42 (37)
Tx	1 (1)
**Nodal status – no. (%)**	
N0	26 (23)
N1	13 (11)
N2	53 (47)
N3	21 (19)

^a^*TPF* docetaxel, cisplatin, 5-fluorouracil

The median weight loss at diagnosis was 4.1 kg. 50% of patients experienced stable weight, 23% weight loss between 5 and 10% from baseline, 19% between 10 and 20 and 8% ≥ 20%. The median serum albumin level was 39.6 g/L (95% CI [37.6–39.8]). Thirty-four patients (30%) had a feeding tube due to initial weight loss.

### TPF induction chemotherapy and treatment following TPF

88% of patients received TPF induction chemotherapy with single-dose cisplatin on D1 and 12% received fractionated cisplatin 20 mg/m^2^ from D1 to D4 from the first treatment. The data concerning TPF induction chemotherapy are presented in Table 3. Sixty-four patients (57%) received the full course of TPF induction chemotherapy as originally planned (without cancellation or dose reduction).

**Table 3:** Therapeutic sequence for the 113 patients treated with TPF induction chemotherapy for locally advanced head and neck cancer

Induction chemotherapy with TPF* regimen	
**Number of cycles – no. (%)**	
4	6 (5)
3	78 (69)
2	19 (17)
1	10 (9)
**Fractionated cisplatin to C1**	
Yes	14 (12)
No	99 (88)
**Patients who received full induction chemotherapy – no. (%)**	70 (62)
**Induction chemotherapy protocol modifications – no. (%)**	
Interruption	18 (16)
Report	7 (7)
Dose reduction	30 (31)
**Radiotherapy**	
**Delay between the end of induction and the start of radiotherapy – median [min-max]**	36 days [24–107]
**Overall treatment time – median [min-max]**	49 days [27–72]
**Technique – no. (%)**	
IMRT*	91 (87)
**Conformational**	13 (13)
**Interruption – no. (%)**	1 (1)
**Concomitant radiochemotherapy (excluding laryngeal preservation and post-operative irradiation)**	
**Concomitant chemotherapy – no. (%)**	**49 (43)**
Carboplatin AUC 1.5/week for 7 weeks	25 (51)
Cisplatin 40 mg/m^2^/week for 7 weeks or cisplatin 100 mg/m^2^ S1, S4, S7	18 (37)
Cetuximab 400 mg/m^2^ at D-8 and 250 mg/m^2^/week for 7 weeks	6 (12)
**Interruptions in concomitant chemotherapy – no. (%)**	26 (53)
Carboplatin	12 46)
Cisplatin	10 (39)
Cetuximab	4 (15)
**Patients who received the full course of treatment – no. (%)**	39 (35%)

*TPF docetaxel, cisplatin, 5-fluorouracil

*IMRT intensity-modulated radiotherapy

The modalities of treatment following TPF induction chemotherapy are presented in Table 3. The median time from the last chemotherapy treatment to the start of radiotherapy was 36 days [24–107] for inoperable LAHNC and for laryngeal preservation. Thirteen percent of patients received conformational technique and 87% received volumetric modulated arc therapy (VMAT). Radiotherapy delivered curative doses on the initial tumor/tumor bed of 60 and 66 Gy for operated patients and 70 Gy for non-operated patients, in fractions of 2 Gy to 2.12 Gy.

Of the 113 patients included, a total of 39 patients (35%) received the full treatment initially planned (induction chemotherapy followed by radiotherapy or radiochemotherapy) without cancellation or dose reduction. Causes for discontinuation of induction chemotherapy or dose reduction were varied: febrile aplasia, acute renal failure, grade ≥ 3 hand-foot syndrome, mucositis, vomiting, diarrhea, radiodermatitis, radiomucitis and death. No patients did not receive concomitant chemotherapy due to induction chemotherapy toxicity.

### TPF induction chemotherapy toxicities and predictive factors of toxicity

TPF induction chemotherapy toxicities are described in Table 4. Predictive factors of these toxicities are described in Table 5.

**Table 4:** Toxicities of induction chemotherapy with TPF in patients treated for locally advanced head and neck cancer. Toxicity was assessed according to CTCAE classification V4.0

	Grade ≥ 3	Grade 4	Grade 5
Anemia	16 (14%)	0 (0%)	0 (0%)
Neutropenia	39 (35%)	32 (34%)	2 (2%)
Febrile neutropenia	30 (27%)	1 (1%)	5 (6%)
Thrombocytopenia	7 (6%)	1 (1%)	1 (1%)
Nausea/Vomiting	24 (22%)	2 (3%)	0 (0%)
Mucositis	20 (18%)	1 (2%)	0 (0%)
Diarrhea	19 (17%)	0 (0%)	0 (0%)
Nephrotoxicity	0 (0%)	0 (0%)	0 (0%)
Digestive hemorrhage	6 (5%)	1 (1%)	1 (1%)

**Table 5:** Predictive factors of toxicities of induction chemotherapy with TPF in patients treated for locally advanced head and neck cancer (multivariate analysis). Toxicity was assessed according to CTCAE classification V4.0

	Anemia	Neutropenia	Febrile neutropenia	Infection	Death
	Grade ≥ 3	Grade ≥ 3	Grade ≥ 3		
Age	NS	NS	NS	NS	NS
Female sex	NS	NS	NS	NS	NS
Alcohol use	NS	NS	NS	NS	NS
Liver dysfunction	NS	NS	NS	NS	NS
Hepatic dysmorphia	NS	NS	NS	NS	NS
Prothrombin rate	**0.049**	NS	NS	NS	NS
Diabetes	NS	NS	NS	NS	NS
HBP	NS	NS	NS	NS	NS
Ischemic heart disease	NS	NS	NS	NS	NS
Obstructive pulmonary disease	NS	NS	NS	NS	NS
Obliterative arteriopathy of the lower limbs	NS	NS	NS	NS	NS
Performance status	NS	NS	NS	NS	NS
Weight loss before TPF	NS	NS	NS	NS	NS
Weight loss during TPF	NS	NS	NS	NS	NS
Enteral feeding during TPF	NS	NS	NS	**0.047**	NS

Indication for laryngeal preservation vs. inoperable disease was not significantly correlated with the different TPF induction chemotherapy toxicities studied (see supplementary data Table S1).

#### Hematotoxicity

14% grade ≥ 3 anemia, 6% grade ≥ 3 thrombocytopenia, 35% grade ≥ 3 neutropenia and 27% grade ≥ 3 febrile neutropenia were obesrved. In univariate analysis, female gender (*p* = 0.042), alcohol consumption (*p* = 0.02), pre-existing liver disease (*p* = 0.037) and a lowered prothrombin rate (< 80%) (*p* = 0.045) were correlated with an increased risk of ≥3 grade anemia. In univariate analysis, age (*p* = 0.039), pre-existing liver disease (*p* = 0.003), and liver dysmorphia on initial imaging (*p* = 0.027) were correlated with an increased risk of grade ≥ 3 thrombocytopenia. No predictive factors for ≥3 grade neutropenia or febrile neutropenia were found. In multivariate analysis, there was an increased risk of ≥3 grade anemia (*p* = 0.049) in patients with a lowered prothrombin rate.

#### Other toxicities

In univariate analysis, female gender was correlated with an increased risk of grade ≥ 3 nausea (*p* = 0.011) and grade ≥ 3 mucositis (*p* < 0.001) and a lowered prothrombin rate at diagnosis (< 80%) was correlated with an increased risk of gastrointestinal bleeding (*p* = 0.03).

In multivariate analysis, there was an increased risk of infection (*p* = 0.047) in patients receiving enteral nutrition during induction chemotherapy. Thirty-six patients (32%) developed an infection during induction chemotherapy.

#### Deaths related to induction chemotherapy

There were 6% deaths (*n* = 7) during TPF induction chemotherapy (3/44 for laryngeal preservation patients, and 4/69 for inoperable patients). Five occurred after the first cycle. Four of them developed febrile neutropenia, one of which was complicated by colitis with gastrointestinal perforation. One patient presented a pneumopathy without neutropenia. One death occurred after the second cycle (sudden cardiopulmonary arrest at home) and another one after the third course (pneumonia in the context of febrile neutropenia). In univariate analysis, an increased risk of death related to induction chemotherapy was found in cases of liver disease (*p* = 0.032), a lowered prothrombin rate at diagnosis (< 80%) (*p* = 0.021) or hepatic dysmorphia on initial imaging (*p* = 0.044). Neither sex, age, tumor stage, location, general condition, weight loss before or during TPF, nor enteral nutrition were significantly predictive of induction-chemotherapy-related death. In multivariate analysis, no factor was significantly correlated with the risk of death.

### TPF induction chemotherapy response rates

Twenty-two percent of patients had a complete response after induction chemotherapy, 60% had a partial response, 12% had a stable disease, 5% had continued progression, and 1% had a discordant response in the primary tumour along with lymphadenopathy.

In univariate analysis, predictive factors of a better response (complete or partial response vs. stable or progressive) were the possibility of oral feeding without a feeding tube (*p* = 0.03), higher albuminemia (*p* = 0.006), and weight loss < 5% at diagnosis (*p* = 0.043). In multivariate analysis, indication for inoperable disease was correlated with better response vs. laryngeal preservation (*p* = 0.04).

### Overall treatment response rates and survival data

The median follow-up was 48 months [0–103], in which 20% of patients had a recurrence (Table 3). Forty-two percent of patients (*n* = 47) were dead at last follow-up. Nine deaths were treatment-related (19%), 7 occurred during induction chemotherapy and 2 during radiotherapy. The median overall survival was 74 months and the median PFS was 20 months.

**Fig. 1:**
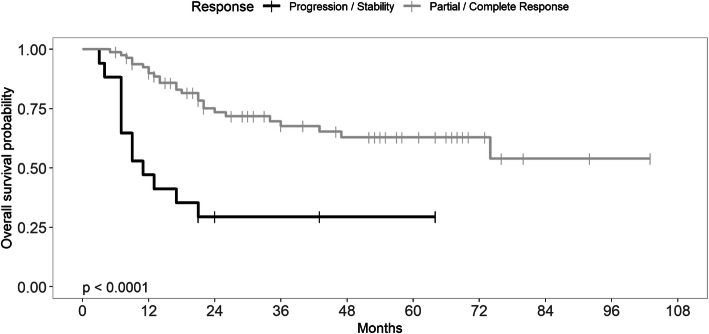
Overall survival of patients treated with TPF induction chemotherapy for locally advanced head and neck cancer based on response to induction chemotherapy

In multivariate analysis, the necessity of enteral feeding at diagnosis and a poor response (stable disease or progression) after induction chemotherapy were predictive of worse PFS (*p* = 0.003 and *p* < 0.001 respectively) and of worse OS (*p* = 0.001 and *p* < 0.001, respectively) (Fig. 1).

## Discussion

TPF induction chemotherapy used in the management of LAHNC has anti-tumor efficacy, with an overall response rate of 82% in our study and from 44 to 81% in the literature for inoperable tumour [8, 20, 22, 23, 30] and about 80% for laryngeal preservation [18]. However, this induction protocol resulted in high rates of severe toxicities as well as a non-negligible rate of death.

The number of patients in our study remains relatively small (113 patients) but represents, to our knowledge, the largest “real life” trial published to date. This study is also retrospective, and thus represents a bias for the interpretation of the results. We included patients receiving TPF induction chemotherapy, either in a laryngeal preservation strategy or for inoperable LAHNC or locally advanced undifferenciated nasopharyngeal carcinoma. This population was therefore heterogeneous, but since the main endpoint of this study was TPF toxicity, this was not a major bias in the interpretation of the results, especially since we have not demonstrated significant differences in toxicities between patients receiving TPF as part of a laryngeal preservation protocol and those receiving TPF for an inoperable tumour. However, efficacy and survival data (secondary endpoints) in our study are to interpret with caution due to the heterogeneity of the population.

We showed that the introduction of an enteral feeding during induction chemotherapy increased the risk of infection (*p* = 0.047 in multivariate analysis). In addition, the prognosis of these patients appears to be generally worse. Indeed, the objective response, progression-free survival and overall survival rates were correlated with the possibility or not of oral feeding at diagnosis. Patients with head and neck cancer are at high risk of undernutrition due to previous alcohol and tobacco intoxication, but also due to impaired swallowing or limited mouth opening that may be caused by the tumor itself [34–36]. This risk may be increased by the mucosal and digestive toxicities specific to TPF. The infectious risks associated with enteral feeding can be of two types: firstly, there is a risk of pneumopathy, either by massive inhalation of nutrient solution or by repeated occult inhalation. Secondly, there is a risk of infection directly related to the placement of the gastrostomy. Our study suggests that patients whose nutritional status is such that they require enteral feeding are not good candidates for TPF induction therapy. Especially for patients with a laryngeal preservation strategy, radiochemotherapy may be more appropriate. However, there was no correlation between the markers of nutritional status used (albuminemia, weight loss) and toxicities, suggesting that these routinely used clinical and biological markers are insufficient. However, concerning albuminemia, our results are to be interpreted with caution since 25% of the data were missing, and in the absence of a systematic C-reactive protein (CRP) dosage. The search for other markers of nutritional status, particularly sarcopenia, would be interesting.

We found 6% iatrogenic deaths in patients receiving TPF induction chemotherapy for LAHNC, a rate similar to the ones found in the literature [8, 20–23, 30]. In our institution, for inoperable LAHNSCC, we reserve TPF induction chemotherapy for patients with rapidly evolving and/or highly symptomatic tumors with a large tumor burden. This is a population similar to that of the GORTEC 2007–02 trial with a death rate of 6.7% [30]. We found that the risk of death was increased in cases of pre-existing liver disease (alcoholic cirrhosis). Liver biology may be normal even in cases of proven cirrhosis and therefore does not by itself allow optimal patient selection. Patients diagnosed with hepatic cirrhosis are usually not treated with TPF, but retrospective analysis of the data from this patient cohort has allowed us to find, a posteriori, signs of cirrhosis prior to chemotherapy in some patients. The extension work-up for these head and neck cancers usually includes a cervico-thoracic CT scan more or less a PET scan for inoperable locally advanced tumors. Therefore, we do not always have at our disposal morphological liver imaging. The systematic performance of an abdominal CT scan or liver ultrasound could be discussed in order not to administer TPF chemotherapy to patients with hepatic dysmorphia.

An option for optimizing induction chemotherapy would be to lighten the chemotherapy regimen to decrease its toxicity, especially for inoperable LAHNC. The use of the modified TPF scheme [37] or the combination of docetaxel, cisplatin and cetuximab, which has achieved response rates of more than 50% [38] with an acceptable safety profile in relapsed or metastatic situations, could be an alternative to TPF. A combination of docetaxel, cisplatin, anti-PD-1 or anti-PD-L1 would also be worth comparing to TPF in terms of the efficacy and safety results of the combination of chemotherapy and anti-PD1 in relapsed or metastatic situations [39].

## Conclusions

The role of TPF induction chemotherapy in the management of locally advanced head and neck cancers remains to be defined. As found in the literature, our study shows that TPF induction chemotherapy has a high objective response rate, but significant morbidity with 6% toxic deaths. Nutritional status and the presence of hepatic dysfunction (liver dysmorphia on imaging or decrease prothrombin rate) seem to be the major elements to be taken into account in therapeutic decisions. Indeed, the need for enteral feeding at the beginning of treatment is associated with an increased risk of toxicities and poorer survival data. The presence of hepatic dysfunction is correlated with an increased risk of hematotoxicity grade ≥ 3 and an increased risk of death.

## Supplementary Information

**Additional file 1: Table S1.** Statistical analysis of toxicity profile differences between the laryngeal preservation group and inoperable disease group.

## Data Availability

Inquiries about datasets analyzed for this study can be directed to the corresponding author.

## Abbreviations

CTCAE: Common Terminology Criteria for Adverse Events
HNSCC: Head and neck squamous cell carcinomas
LAHNC: Locally advanced head and neck cancers
OS: Overall survival
PF: Cisplatin-5-fluorouracil
PFS: Progression-free survival
RECIST: Response evaluation criteria in solid tumours
RT: Radiotherapy
TPF: Docetaxel-cisplatin-5-fluorouracil
VMAT: Volumetric modulated arc therapy
