# Supplementary material for: Predictive factors of toxicity of TPF induction chemotherapy for locally advanced head and neck cancers
Source: BMC Cancer. 2021 Apr 7;21:360. doi: 10.1186/s12885-021-08128-5 (PMC8025378; doi:10.1186/s12885-021-08128-5)
Supplement: Supplementary file 1 — Additional file 1: Table S1. Statistical analysis of toxicity profile differences between the laryngeal preservation group and inoperable disease group. [file 12885_2021_8128_MOESM1_ESM.docx]

**Table S1. Statistical analysis of toxicity profile differences between the laryngeal preservation group and inoperable disease group**

|  | Laryngeal preservation vs. inoperable disease | | |
| --- | --- | --- | --- |
| Toxicities | RR | 95% Ci | p |
| Anemia | 0.45 | (0.17 – 1.11) | 0.164 |
| Neutropenia | 0.78 p | (0.47 – 1.29) | 0.423 |
| Febrile neutropenia | 0.77 | (0.41 – 1.43) | 0.52 |
| Thrombocytopenia | 0.22 | (0.04 – 1.06) | 0.106 |
| Nausea | 1.11 | (0.51 – 2.36) | 0.644 |
| Vomiting | 1.52 | (0.44 – 5.21) | 0.364 |
| Mucositis | 0.83 | (0.37 – 1.86) | 0.805 |
| Diarrhea | 0.51 | (0.22 – 1.15) | 0.194 |
| Nephrotoxicity | 0.37 | (0.08 – 1.55) | 0.42 |
| Digestive hemorrhage | 0.85 | (0.16 – 4.45) | 0.99 |
